# Evaluation of the Safety and Effectiveness of Intense Pulsed Light in the Treatment of Meibomian Gland Dysfunction

**DOI:** 10.1155/2016/1910694

**Published:** 2016-06-20

**Authors:** Xiaodan Jiang, Huibin Lv, Hang Song, Mingzhou Zhang, Yan Liu, Xiaodan Hu, Xuemin Li, Wei Wang

**Affiliations:** Ophthalmology Department, Peking University Third Hospital, Beijing 100191, China

## Abstract

*Purpose.* This study aims to explore the safety and efficacy of a novel treatment-intense pulsed light (IPL) in MGD eyes.* Methods.* This study is a prospective and open label study. Forty eyes of 40 MGD patients were recruited in the study and received 4 consecutive IPL treatments on day 1, day 15, day 45, and day 75. Ten ocular surface symptoms were evaluated with a subjective face score at every visit. Best spectacle corrected visual acuity, intraocular pressure (IOP), conjunctival injection, upper and lower tear meniscus height (TMH), tear break-up time (TBUT), corneal staining, lid margin and meibomian gland assessments, and meibography were also recorded at every visit, as well as the adverse effects on the eye and ocular surface.* Results.* Significant improvements were observed in single and total ocular surface symptom scores, TBUT, and conjunctival injection at all the visits after the initial IPL treatment (*P* < 0.05). Compared to baseline, the signs of eyelid margin, meibomian gland secretion quality, and expressibility were significantly improved at every visit after treatments. There was no regional and systemic threat observed in any patient.* Conclusion.* Intense pulsed light (IPL) therapy is a safe and efficient treatment in relieving symptoms and signs of MGD eyes.

## 1. Introduction

Meibomian Gland Dysfunction (MGD) is one of the most common causes of dry eye [1]. It is a diffuse deformity of the meibomian glands, whose terminal duct is fully or partly obstructed. The glandular secretion is changed in quality or/and quantity [1], which results in an unstable tear film. Its main symptoms range from dryness, eye irritation, foreign body sensation, burning, and watering to fatigue [2]. The prevalence of MGD varies broadly worldwide, from 3.5% to nearly 70% [3], which is of concern to clinical doctors and scientists.

The pathogenesis of MGD begins with ductal epithelium hyperkeratinization and increased meibum viscosity. The obstruction subsequently happens when the terminal duct is filled with thickened opaque meibum which contains keratinized cell material, resulting in intraglandular cystic dilation, gland dropout, and low secretion [4, 5]. Reduced meibum outflow will boost the proliferation of commensal bacteria [6, 7], releasing fatty acids and mono- and diglycerides into the tear film causing a sense of irritation [8, 9].

The treatments of MGD vary from artificial tears, warm compression [10–13], meibomian gland expression [14–16], and omega-3 supplementation [17] to cyclosporine [18], corticosteroids, and oral antibiotics [19], all of which have been shown to provide only short-term symptom relief [2, 20]. This suggests that we need more treatment options, one of which is intense pulsed light therapy. Intense pulsed light (IPL) therapy is generally used in the cosmetic industry [21] for disease like benign cavernous hemangiomas, telangiectasia, port-wine stains, and so forth [21–25]. IPL treatment applies Xenon flash lamp to emitting wavelengths of light ranging from 400 to 1200 nm, and various chromophores (hemoglobin, melanin, and water) will be targeted concurrently [26].

The initial application of intense pulsed light for dry eye patients began in 2002 by Dr. Rolando Toyos when a patient with rosacea indicated improvement of dry-eye symptoms after receiving IPL treatment [27–30]. Since then, studies about the treatment of dry eye syndrome caused by MGD by IPL have gradually shown its benefits [31, 32]. The prevalence of MGD in Asian populations (>60%) is much higher than that of Caucasians (3.5%–19.9%) [4]. Due to the differences of the pigmentation and skin type between Chinese and Caucasian, the efficacy and safety of the IPL wavelengths might be diverse. While similar studies in Chinese patients in this field are rather few, the efficacy, safety, and mechanism of this new therapy in Chinese patients needs further evaluation.

Our study aimed to collect the data of IPL therapy in Chinese MGD patients so that we can clarify the effectiveness and safety of IPL therapy in Chinese MGD patients. The IPL device we used is currently the only certified IPL device (E-Eye; E-SWIN, Paris, France) for treating MGD [31]. The wavelength of the device ranges from the lower visible spectrum (580 nm) to near infrared (1200 nm).

## 2. Materials and Methods

### 2.1. Patients

The present study was conducted according to the principles of the Declaration of Helsinki and was approved by the Human Research and Ethics Committee of Peking University Third Hospital. Written informed consent form was obtained from each participant before enrolment. This study is a prospective and open label study.

Subjects were recruited from the outpatient department of the Department of Ophthalmology of Peking University Third Hospital between April 2014 and January 2015. The inclusion criteria for this study were (1) adult patients; (2) chief complaint of one of the following symptoms: dryness, foreign body sensation, burning, and tearing for more than 3 months; (3) diagnosis of MGD with two or more of the following signs in both eyes: redness or thickening of the lid margin, telangiectasia, reduced or no secretions, poor quality secretions, and gland capping [33]; (4) willingness to cooperate with the doctors in the follow-up visits. Exclusion criteria included patients with (1) severe ocular surface abnormalities; (2) history of ocular trauma or surgery; (3) punctal occlusion; (4) use of any eye drops other than artificial tears within the past 1 month; (5) active allergy or infection or inflammatory disease at the ocular surface unrelated to dry eye or MGD; (6) current use of treatments for MGD; (7) alterations of the lacrimal drainage system, (8) use of systemic medications altering the tear film; (9) contact lens wear; (10) systemic diseases affecting the ocular surface; (11) uncontrolled systemic disease; (12) pigmented lesions in the treatment area; (13) skin treatments within 2 months; (14) pregnancy/nursing mothers.

Forty eyes (left eyes were selected at random) of 40 MGD patients (18 males and 22 females) were enrolled into this prospective study, with a mean age of 51.3 ± 20.1 years (ranged 21–78 years).

### 2.2. Treatment Procedure

With an E-Eye machine provided by E-SWIN company, France (https://www.e-swin.com/), IPL treatment was administered to the skin area below the lower eyelid [31]. Before treatment, the eyes were protected with opaque goggles and ultrasound gel was applied on the patient's face from tragus to tragus including the nose to conduct the light, help to spread the energy evenly, and provide a degree of protection [32]. The intensity of the IPL treatment ranges from 9.8 J/cm^2^ to 13 J/cm^2^ in accordance with the Fitzpatrick Skin Type Grading [31]. In each IPL treatment, 4 overlapping flashes were applied to the skin area below the lower eyelid for every eye with no pressure. All the treatment areas were identical within different subjects. The subjects received four separate treatment sessions on day (D) 1, D15, D45, and D75. All the treatments were performed by the same doctor (JXD).

### 2.3. Clinical Evaluation

Subjects were evaluated at four visits: 3 days before the first IPL treatment and 15 days, 45 days, 75 days after the first treatment. The clinical assessments of the subject and both eyes were carried out in the following order: symptoms evaluation, best spectacle corrected visual acuity, intraocular pressure (IOP), conjunctival injection, upper and lower tear meniscus height (TMH), tear break-up time (TBUT), corneal staining, lid margin and meibomian gland assessments, and meibography. An interval of 5 minutes was required between different tests. All the test data were collected by two doctors (LHB and ZMZ); the average would be defined as the final results. All the evaluations and assessments were carried out before the IPL treatment at each visit.

### 2.4. Symptom Evaluation

The severity of the following 10 ocular symptoms for every subject was assessed by the ophthalmologist at clinic visits on baseline, day 15, day 45, and day 75: dryness, foreign body sensation, watering, itchiness, visual fatigue, blurred vision, burning, sensitivity to light, secretion disturbance, and pain. For each symptom assessment, subjective face scores were applied [20]. 11 faces were shown to the subjects, with the saddest face (score 10) representing the most severe discomfort and the happiest face (score 0) describing no discomfort. A total subjective symptom score was defined as the summation of these scores; thus minimum of score was 0, and maximum was 100.

### 2.5. Intraocular Pressure (IOP)

IOP of every eye was measured by noncontact tonometer (Canon TX-20, Japan) with no topical anaesthetic used, which is not invasive. The procedure was evaluated for three times, and the average value was defined as the final score.

### 2.6. Conjunctival Injection and TMH

Conjunctival injection degree was evaluated under slit lamp microscope. Institute for Eye Research (IER) Grading Scales [34] were used to assess bulbar redness with score 0 representing grade 1 and score 3 meaning grade 4, which describe severe redness of the bulbar conjunctiva. The central upper and lower TMH were measured by a slit lamp microscope (with a graticule in 0.05 mm units) [35]. Three consecutive readings were evaluated and the median was defined as the final results.

### 2.7. TBUT and Corneal Staining

A total of 5 *μ*L of 2% sodium fluorescein was instilled onto the bulbar conjunctiva without inducing reflex tearing, by using a micropipette [36]. The patient was asked to blink naturally without squeezing for three to five times, and then the patient was asked to stare straight ahead without blinking, until told otherwise, under the cobalt blue light [36]. A stopwatch was used to record the time between the last complete blink and the first appearance of a dry spot or disruption in the tear film [36]. The procedure was evaluated for three times, and the average value was defined as the final score. For corneal staining evaluation, the cornea was divided into five sectors [37]; each sector was graded from 0 to 3 using the following criteria: 0 no staining; 1 punctate/stipple staining; 2 ball and linear staining; and 3 coalesced staining [38].

### 2.8. Eyelid Margin and Meibomian Gland Assessments

According to the International Workshop on Meibomian Gland Dysfunction, five signs of eyelid margin were assessed in our study: rounding of posterior margin, irregularity/notching of margin, telangiectasia/vascularity of lid margin, trichiasis, and anterior blepharitis. Each sign scored 0 or 1. Score 0 equals no/normal; score 1 equals yes/abnormal. Meibomian gland assessments included (1) the average of the number of the upper and lower present lid orifices; (2) expressed secretion quality; (3) the expressibility of the meibomian gland. The levels of the quality were divided into 4 degrees: 0 = clear; 1 = cloudy; 2 = granular; 3 = toothpaste, as well as the expressibility: 1 = light; 2 = moderate; 3 = heavy pressure [39].

### 2.9. Safety Evaluation

At every visit, best spectacle corrected visual acuity, intraocular pressure (IOP), and corneal and conjunctival examinations by slit lamp microscope were performed. Eyelash abnormities such as eyelash loss and trichiasis were evaluated by slit lamp microscope. The assessments of the skin area around the eye were also carried out for the examination of depigmentation, blistering, swelling, redness, and hair loss at brow and forehead.

### 2.10. Statistical Analysis

Statistical analysis was performed by using R software (Version 2.14.2). Comparison between data points was performed with paired *t*-test with the Bonferroni correction, which compared the single and total symptoms, conjunctival injection, TMH, TBUT, corneal staining, and the number of meibomian gland orifices at the three different follow-up times to those of the last visits. Chi square test was used to compare the eyelid margin signs, meibomian gland secretions, and expressibility at three different follow-up times to those of last visits.

## 3. Results

The full cohort of 40 enrolled participants completed measurements across all four appointments and were included in the analysis.

### 3.1. Clinical Symptoms

**Figure 1:**
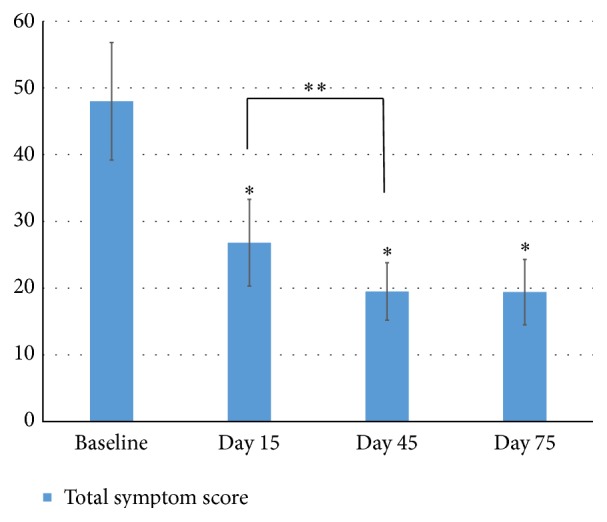
Total symptom scores. Notes: the total scores of the 10 single symptoms were defined as the total symptom scores. ^*∗*^ Compared to the baseline, the total symptom score significantly decreased at the time of D15, D45, and D75 (*P* < 0.01). ^*∗∗*^ Between the visits of D15 and D45, the total score continuously decreased (*P* = 0.04), while between the visits of D45 and D75, no significant difference was observed (*P* = 1). Statistical analysis was performed with paired *t*-test with the Bonferroni correction.

Ten ocular surface symptoms were evaluated at every visit and the results were listed in Table 1. The symptom scores were collected before the treatment with IPL at every visit. Compared to baseline, all symptoms were significantly relieved at the time of D15, D45, and D75 (*P* < 0.05) except blurred vision. Between the visits of D15 and D45, significant relief was continuously observed in dryness (*P* < 0.01) and pain (*P* = 0.03). However, between the visits of D45 and D75, there was no significant difference among all symptoms. The total score of the 10 single symptoms was defined as the total symptom scores (Figure 1). Compared to baseline, the total symptom score significantly decreased at the time of D15, D45, and D75 (*P* < 0.01). Between the visits of D15 and D45, the total score continuously decreased (*P* = 0.04), while between the visits of D45 and D75, no significant difference was observed (*P* = 1).

### 3.2. Eyelid Margin and Meibomian Gland Assessments

**Figure 2:**
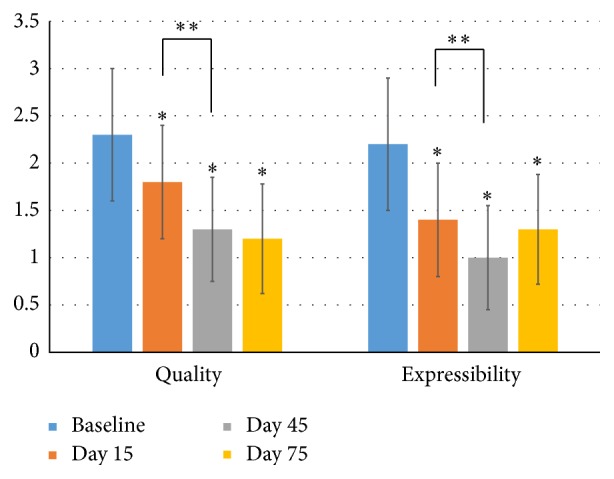
Meibomian gland secretion quality and expressibility. Notes: the *y* axis represents the level or grade of the meibomian gland secretion quality and expressibility, with higher grades meaning worse quality and expressibility. ^*∗*^ Compared to the baseline, the meibomian gland secretion quality and expressibility significantly improved at the visits of D15, D45, and D75 (*P* < 0.05). ^*∗∗*^ Between the visits of D15 and D45, the meibomian gland secretion quality and expressibility continuously improved (*P* < 0.05). Between the visits of D45 and D75, no significant difference was observed for the secretion quality (*P* = 0.68) and expressibility (*P* = 0.29). Statistical analysis was performed with paired *t*-test with the Bonferroni correction.

Five signs of eyelid margin, rounding of posterior margin, irregularity/notching of margin, telangiectasia/vascularity of lid margin, trichiasis, and anterior blepharitis, were evaluated at every visit and recorded in Table 2. Compared to baseline, all the signs except trichiasis were significantly improved after the treatments (*P* < 0.05). The number of the meibomian gland orifices within the central 1 cm was significantly increased at D15 (4.3 ± 3.1), D45 (5.3 ± 3.5), and D75 (4.9 ± 3.3), compared to those from baseline (*P* < 0.01). Between the visits of D15 and D45, the number continuously increased (*P* = 0.02), while between the visits of D45 and D75, no significant difference was observed (*P* = 0.96). Compared to the baseline, the meibomian gland secretion quality and expressibility significantly improved at the visit of D15 (*P* < 0.05) and continuously improved at the visit of D45, which was compared to those at D15 (*P* < 0.05) (Figure 2). Between the visits of D45 and D75, no significant difference was observed for the secretion quality (*P* = 0.68) and expressibility (*P* = 0.29) (Figure 2).

### 3.3. TBUT and Corneal Staining

**Figure 3:**
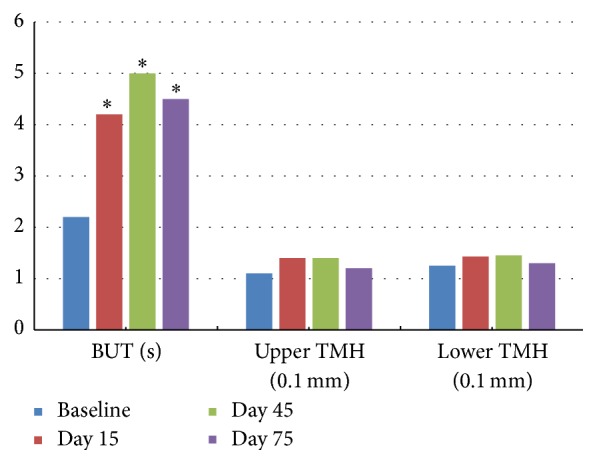
Tear break-up time (TBUT) and tear meniscus height (TMH). Notes: ^*∗*^ TBUT at D15 (4.2 ± 1.8 s), D45 (5.0 ± 1.9 s), and D75 (4.5 ± 2.5 s) were significantly increased compared to that at the baseline (2.2 ± 1.5 s) (*P* < 0.01). Between the visits of D15 and D45, TBUT continuously increased while reaching no statistic difference (*P* = 0.07). Between the visits of D45 and D75, no significant difference was observed (*P* = 0.51). No significant difference was found in the assessment of upper and lower TMH in all visits. Statistical analysis was performed with paired *t*-test with the Bonferroni correction.

TBUT at D15 (4.2 ± 1.8), D45 (5.0 ± 1.9), and D75 (4.5 ± 2.5) were significantly increased compared to that at the baseline (2.2 ± 1.5) (*P* < 0.01) (Figure 3). Between the visits of D15 and D45, TBUT continuously increased while reaching no statistic difference (*P* = 0.07). Between the visits of D45 and D75, no significant difference was observed (*P* = 0.51). No significant difference was found in the assessment of corneal staining among all visits (Table 3).

### 3.4. Conjunctival Injection and TMH

Compared to baseline, conjunctival injection was significantly relieved at D15, D45, and D75 (*P* = 0.01). Among the visits of D15, D45, and D75, no significant difference was observed (Table 3). No significant difference was found in the assessment of upper and lower TMH among all visits (Figure 3).

### 3.5. Safety Data

Among all visits, best spectacle corrected visual acuity was not significantly changed; IOPs of all subjects were lower than 21 mmHg. There was no depigmentation, blistering, swelling, redness, and hair loss at the brown and ocular surface. There was no significant eyelash loss during the evaluation, either. No systemic adverse event was observed during the study.

## 4. Discussion

Meibomian Gland Dysfunction (MGD) is a high prevalent ocular surface disease. The efficacy of conventional treatment for MGD remains to be transient and unsatisfactory, suggesting the need for the exploration of new therapeutic approaches. Our study applied IPL treatment to the skin around the eyes in 40 Chinese MGD patients (40 eyes) and provided a strong evidence for the effectiveness and safety of IPL treatment in relieving ocular surface symptoms and signs.

In our research, a series of 10 comprehensive subjective self-reported symptoms associated with MGD were evaluated with a face scorecard. All symptoms except blurred vision significantly improved after the initial IPL treatment. Furthermore, two symptoms, dryness and pain, continuously relieved significantly after the second IPL treatment, as well as the total symptom scores. The improvement of the single and total symptom scores remains steady after the visits of D45 to D75, which implied that twice IPL treatments might meet the maximum therapeutic effects in relieving ocular surface symptoms. For a long time, MGD associated dry eye has been also considered as a chronic pain disease [38]. Much research indicates that chronic inflammatory status of MGD or dry eye is able to lower the pain threshold and increase neurogenic sensitivity through proinflammatory factors [40]. Wavelength of 600–950 nm which is included in our IPL treatment is proved to be effective in relief of inflammation pain and neurogenic sensitivity [41, 42]. Our study also showed that the ocular pain was relieved significantly after the IPL treatment. One of the mechanisms of symptoms relief effect may relate to the neurogenic sensitivity and pain adjustments of IPL therapy. Blurred vision in MGD and dry eye patients are mainly caused by shortened BUT, reaching no significant change in our study. Even though the BUT increased after IPL treatment, the BUT at every visit was still far away from normal (>10 s), which may resulted in the blurred vision symptom. On the other hand, the blurred vision symptom did not aggravate which implied the safety profile that the IPL treatment exerted no influence on the vision acuity.

Five signs of the eyelid margin: rounding of posterior margin, irregularity/notching of margin, telangiectasia/vascularity of lid margin, trichiasis, and anterior blepharitis were evaluated in our study. Rounding of posterior margin, irregularity, telangiectasia, and anterior blepharitis experienced great improvements. Numerous studies showed that hemoglobin primarily absorbs at a wavelength of 580 nm [43] and then causes the blood cells in the abnormal telangiectasias to absorb the light, to coagulate, and, finally, to close the blood vessels, thus improving vascularization [32]. Our study showed similar effects of IPL treatment: eyelid telangiectasia were significantly relieved, as well as the conjunctival injection. Such improvements in telangiectasia may prevent inflammatory mediator secretion and decrease bacterial overgrowth [32].

The alteration of meibomian gland secretion quality and expressibility is the key characteristics in MGD eyes. Our research revealed significant improvements of meibomian gland secretion quality and expressibility after IPL treatments. Similar results were observed in Goto et al.'s research [20], who applied an infrared warm compression device to the meibomian gland. Studies [3] showed that melting point of meibomian gland secretions in subjects with MGD was 3°C higher than that in normal eyes and thermal therapies such as warm compression were able to melt the pathologically dysfunctional lipids and relieve the ocular surface symptoms associated with MGD. Theoretically, the light coming from IPL device is directly exposed to the skin and could result in a production of heat higher than body temperature [31], which is enough to melt the pathological secretion. During the IPL treatment, enough ultrasound gel should be used on the patient's face from tragus to tragus including the nose to conduct the light, help to spread the energy evenly, and provide a degree of protection.

Our study also found that TBUT was significantly lengthened after IPL treatment. Tear film is a highly organized structure on the ocular surface; its stability and function are highly relied on its biochemical composition [44]. The improvement of the meibomian gland secretion quality and expressibility by IPL treatment may have a direct effect on the stability of tear film. Craig and colleagues [31] found out that IPL therapy was able to improve the lipid layer grade in tear film. The presumed decrease of proinflammatory factors arising from the decreased the eyelid telangiectasia and conjunctival injection relief observed in our study may also play a role in the stability of tear film.

Overall, there are some possible mechanisms whereby IPL treatment could relieve ocular surface symptoms and signs of MGD eyes. First, IPL is able to produce a heat effect which melts the pathologically dysfunctional secretions. Second, the IPL device we applied emits energy in a band from a base of the visible spectrum (580 nm) to near infrared (1200 nm), which can be absorbed by hemoglobin, causing the thrombosis of the abnormal vascular in eyelid margin and related conjunctiva. Third, IPL treatment may exert an effect in relief of inflammation pain and neurogenic pain [41, 42], which is highly related to the improvement of clinical symptoms.

There are some limitations in our study including the following. (1) The first is lack of control group. The compressing effect of the goggles worn during the IPL may exert a role, which should be ruled out; lacking a nontreatment control group, the placebo effect and the risk of investigator bias could have influenced the results. Further studies should be carried out with placebo controls or positive controls to rule out the above influences. (2) The second is reporting subjective symptoms in an open label study. Reporting subjective symptoms which would be considered a low level of scientific evidence was applied in our study. (3) The short time of observation was limited to 75 days; the final treatment was performed at the very day as the final evaluation which suggested that the full effect of the final treatment might not have been realised. A follow-up is also needed after treatment termination to assess the long-term effectiveness and safety of such treatment. (4) TMH measurement technique is too insensitive (with a graticule in 0.05 mm units) to reveal differences. A better technique should be applied for better sensitivity. (5) Mechanisms of IPL treatment in MGD eyes were not proven in our study. Further research should be carried out to explore the exact mechanisms or molecular changes during IPL therapy in MGD eyes.

## 5. Conclusion

Meibomian Gland Dysfunction (MGD) is one of the most common causes of dry eye, resulting in a range of symptoms including dryness, burning, foreign body sensation, and blurred vision. The treatments of MGD, which are numerous, remain to be inefficient and not comprehensive. IPL treatment is a newly advanced choice for MGD patients. Our study applied a consecutive IPL treatment to Chinese MGD patients and demonstrated that it was able to relieve the symptoms and signs of MGD in Chinese patients safely and effectively, which may open up a potential new treatment for MGD.

## Figures and Tables

**Table 1:** 10 ocular surface symptoms' evaluation during IPL treatments.

Symptoms	Mean ± SD						
	Baseline	D15	*P* ^*∗∗*^	D45	*P* ^*∗∗∗*^	D75	*P* ^*∗∗∗∗*^
Dryness	9.5 ± 2.2	5.9 ± 3.5	<0.01^*∗*^	4.1 ± 3.1	<0.01^*∗*^	4.2 ± 3.0	1
Foreign body sensation	8.0 ± 4.1	4.8 ± 3.9	<0.01^*∗*^	3.5 ± 3.4	0.129	3.7 ± 3.2	1
Itching	3.8 ± 4.9	1.8 ± 3.4	0.03	1.1 ± 2.6	0.54	1.0 ± 2.6	1
Burning	3.8 ± 4.9	1.1 ± 2.8	<0.01^*∗*^	0.9 ± 2.5	1	1.3 ± 2.8	0.93
Visual fatigue	5.0 ± 5.1	3.5 ± 4.0	0.01^*∗*^	2.8 ± 3.4	0.24	2.5 ± 3.4	1
Blurred vision	1.0 ± 3.1	0.7 ± 2.2	0.54	0.3 ± 1.5	1	0.2 ± 0.9	1
Sensitivity to light	4.3 ± 5.0	2.3 ± 3.4	<0.01^*∗*^	1.8 ± 3.1	0.45	1.6 ± 2.8	1
Watering	2.5 ± 4.4	1.1 ± 2.8	0.02^*∗*^	0.8 ± 2.4	1	0.7 ± 1.9	1
Secretion disturbance	5.8 ± 5.0	2.8 ± 3.3	<0.01^*∗*^	2.7 ± 3.4	1	2.6 ± 3.8	1
Pain	4.3 ± 5.0	2.8 ± 3.9	0.04^*∗*^	1.5 ± 2.7	0.03^*∗*^	1.6 ± 3.1	1

Notes: ^*∗*^ *P* < 0.05; ^*∗∗*^compared to baseline; ^*∗∗∗*^compared to D15; ^*∗∗∗∗*^compared to D45. Statistical analysis was performed with paired *t*-test with the Bonferroni correction.

SD: standard deviation; D: day.

**Table 2:** Evaluation of eyelid margin signs during IPL treatments.

Eyelid margin	*n* (%)				*P* ^*∗∗*^
	Baseline	Day 15	Day 45	Day 75	
Rounding of posterior margin	29 (72.5%)	22 (55.0%)	16 (40.0%)	14 (35.0%)	<0.01^*∗*^
Irregularity	23 (57.5%)	12 (30.0%)	6 (15.0%)	6 (15.0%)	<0.01^*∗*^
Telangiectasia	26 (65.0%)	20 (50.0%)	11 (27.5%)	6 (15.0%)	<0.01^*∗*^
Trichiasis	1 (2.5%)	1 (2.5%)	0 (0%)	3 (7.5%)	0.27
Anterior blepharitis	38 (95.0%)	35 (87.5%)	30 (75.0%)	28 (70.0%)	0.01^*∗*^

Notes: ^*∗*^ *P* < 0.05; ^*∗∗*^Chi square test.

**Table 3:** Evaluation of number of orifices within central 1 cm, conjunctival injection, and corneal staining changes during IPL treatments.

	Mean ± SD			
	Baseline	Day 15	Day 45	Day 75
Number of orifices (central 1 cm)	2.8 ± 2.8	4.3 ± 3.1	5.3 ± 3.5	4.9 ± 3.3
*P* ^*∗∗*^		<0.01^*∗*^	0.02^*∗*^	0.96
Conjunctival injection	0.95 ± 0.45	0.60 ± 0.55	0.58 ± 0.55	0.63 ± 0.49
*P* ^*∗∗*^		0.01^*∗*^	1	1
Corneal staining	0.15 ± 0.49	0.03 ± 0.16	0.03 ± 0.16	0.08 ± 0.35
*P* ^*∗∗*^		0.39	1	0.96

Notes: ^*∗*^ *P* < 0.05; ^*∗∗*^paired *t*-test compared to the previous visit with the Bonferroni correction.

SD: standard deviation.
